# Einfluss von lokalen Therapiemaßnahmen auf die Biologie des fortschreitenden Prostatakarzinoms: Die Behandlung des Primärtumors kann die Hormonresistenz der Metastasen verzögern

**DOI:** 10.1007/s00120-022-01788-6

**Published:** 2022-03-08

**Authors:** Johannes Linxweiler, Turkan Hajili, Matthias Saar, Christina Maßmann, Kerstin Junker, Michael Stöckle

**Affiliations:** 1 Klinik für Urologie, Universität des Saarlandes, Kirrberger Str., 66421 Homburg/Saar, Deutschland; 2 Urologische Klinik, Diako Krankenhaus Flensburg, Flensburg, Deutschland; 3 Klinik für Urologie, Universitätsklinikum RWTH Aachen, Aaachen, Deutschland

**Keywords:** Lokal fortgeschrittenes Prostatakarzinom, Radikale Prostatektomie, Kastrationsresistenz, Hormontherapie, Induktive Therapie, Locally-advanced prostate cancer, Radical prostatectomy, Castration resistance, Hormonal therapy, Inductive therapy

## Abstract

**Hintergrund:**

In den letzten 15 Jahren zeigt sich ein Trend hin zu einem längeren Überleben beim metastasierten Prostatakarzinom. Neben dem durch neue Medikamente bedingten Fortschritt deuten retrospektive Daten auch auf einen möglichen positiven Effekt einer früheren Primärtumorbehandlung hin.

**Fragestellung:**

Kann eine Primärtumorbehandlung im Falle einer späteren Metastasierung die Prognose der betroffenen Patienten verbessern und wenn ja, über welche Mechanismen?

**Material und Methode:**

Wir werteten die klinischen Langzeitergebnisse von 115 Patienten aus, die bei T4-Prostatakarzinomen nach induktiver Hormontherapie an unserer Klinik prostatektomiert worden waren. Weiterhin erfolgte eine kritische Durchsicht und Diskussion der zur oben genannten Fragestellung vorhandenen Literatur.

**Ergebnisse:**

Von den 115 Patienten hatten 84 im weiteren Verlauf ein biochemisches Rezidiv erlitten, waren also definitiv durch die radikale Prostatektomie nicht geheilt. Das tumorspezifische und das Gesamtüberleben dieser 84 Patienten lag nach 150 Monaten bei 61 % bzw. 44 %. Bemerkenswert war die Beobachtung, dass diese Patienten ein überraschend gutes und langes Ansprechen auf eine Hormontherapie zeigten. Von den 84 Patienten waren nach durchschnittlich 95 Monaten Nachbeobachtungszeit noch 47 am Leben. 31 von ihnen, also ungefähr zwei Drittel, standen immer noch unter einer Standardhormontherapie. Nur 13 hatten eine Resistenz gegen die primäre Hormontherapie entwickelt und entsprechend eine tertiäre Hormontherapie erhalten, auf die sie teilweise aber auch wieder langfristig sensibel blieben.

**Schlussfolgerungen:**

Die Primärtumorentfernung, zumindest unter den beschriebenen Begleitumständen, scheint die Entwicklung einer Hormonresistenz beim metastasierten Prostatakarzinom hinauszögern und in Einzelfällen sogar ganz verhindern zu können.

## Abstract (en)

**Background:**

In metastatic prostate cancer, a trend towards longer survival has been observed over the last 15 years. Beyond progress due to new drugs, retrospective data also suggest a positive influence of a prior treatment of the primary tumor.

**Objectives:**

Can treatment of the primary tumor improve the prognosis of patients later developing metastases, and if yes, what are the underlying mechanisms.

**Materials and methods:**

In addition to a critical review and discussion of the literature, we analyzed the long-term outcomes of 115 patients with T4 prostate cancer, who had undergone radical prostatectomy after inductive hormonal therapy at our institution.

**Results:**

Of the 115 patients, 84 developed prostate-specific antigen (PSA) recurrence during the further course of disease and must therefore be regarded as uncured. Tumor-specific and overall survival of these 84 patients after 150 months were 61 and 44%, respectively. A total of 47 patients were alive after a median follow-up time of 95 months, of whom 31 were still receiving standard hormonal therapy. Only 13 had developed resistance towards their primary hormonal therapy and, hence, received tertiary hormonal therapy. Again, long-term responses were found in some of these patients.

**Conclusions:**

Primary tumor resection, at least under the circumstances described here, seems to delay the development of castration resistance in metastatic prostate cancer or to completely prevent it in individual cases.

## Einleitung

Die Erstbeschreibung der Hormonabhängigkeit des Prostatakarzinoms erfolgte durch Huggins und Hodges 1941 und wurde 1966 mit dem Nobelpreis für Medizin und Physiologie ausgezeichnet [15]. Hierdurch wurde die Androgendeprivationstherapie (ADT) – zunächst als chirurgische Kastration, später durch LHRH-Agonisten und -Antagonisten abgelöst – zum Therapiestandard für das metastasierte Prostatakarzinom, dessen natürlichen Krankheitsverlauf unter dieser Therapieform man für Jahrzehnte als weitestgehend unbeeinflussbar ansah. Noch 2002 haben Eisenberger und Carducci das scheinbare Ende der hormontherapeutischen Möglichkeiten mit diesen Worten zusammengefasst: „The development of a hormone-independent state is a categorical and irreversible phenomenon observed in the majority of patients and occurs within an almost predictable time frame after the initiation of androgen deprivation“ [10]. Bis zu diesem Zeitpunkt zeigten alle verfügbaren Studiendaten in guter Übereinstimmung unter ADT eine mediane Zeit bis zur Progression von 12 bis 18 Monaten und bis zum tumorbedingten Tod von 2 bis 3 Jahren. Auch die Einführung von Erstgenerationsandrogenrezeptorantagonisten wie Flutamid oder Bicalutamid, oft als sekundäre Hormontherapie bezeichnet, konnte diese Überlebenszeiten nicht signifikant verbessern [2, 25].

Diese deprimierend schlechte Prognose hat sich in den letzten beiden Jahrzehnten jedoch durch zwei Einflussfaktoren wesentlich verbessert: Zum einen durch eine Vielzahl neuer Medikamente, die allesamt gezeigt haben, dass sie die Überlebenszeit der betroffenen Patienten signifikant verlängern können [4, 5, 8, 26]. Zum anderen wurde seit Beginn des jetzigen Jahrhunderts auch ohne direkten medikamentösen Einfluss wiederholt ein verlängertes Überleben bei metastasiertem Prostatakarzinom beschrieben, wenn vor dem Auftreten der Metastasen eine Primärtumorbehandlung stattgefunden hatte. Die ersten, die auf dieses Phänomen aufmerksam gemacht haben, waren Thompson et al., die 2002 in einer Ex-post-Analyse der SWOG-8849-Studie (Orchiektomie + Placebo vs. Orchiektomie + Flutamid) zeigen konnten, dass Patienten, die vor Eintritt der Metastasierung prostatektomiert worden waren, länger gelebt haben als Patienten, deren Metastasen ohne Vorbehandlung des Primärtumors aufgetreten waren [28]. In den Folgejahren gab es eine Reihe weiterer Hinweise, dass die Behandlung des Primärtumors Einfluss auf die Biologie der Metastasen und damit den Verlauf der metastasierten Erkrankung haben könnte: So berichteten Qin et al. 2012, dass eine palliative TURP das Überleben von Patienten mit metastasiertem Prostatakarzinom verlängert [24]. Shao et al. publizierten 2014 eine SEER-Datenbankanalyse, die neuerlich bei Patienten mit metastasiertem Prostatakarzinom eine Überlebensverlängerung zeigte, wenn bei den Patienten vor Auftreten von Metastasen eine radikale Prostatektomie durchgeführt worden war [27].

Vor dem Hintergrund dieser Beobachtungen möchten wir im Folgenden die Ergebnisse aus unserer eigenen Klinik zur Fragestellung eines positiven Einflusses einer Primärtumortherapie auf die Prognose des metastasierten Prostatakarzinoms vorstellen. Es handelt sich hierbei um die Zusammenfassung zweier kürzlich publizierter Originalarbeiten [13, 22], welcher wir eine Diskussion der möglicherweise zugrunde liegenden molekularen Mechanismen anschließen.

## Ergebnisse

Bei der Analyse einer eigenen Serie von 115 Patienten, die bei einem initial inoperablen T4-Prostatakarzinom nach induktiver Hormontherapie zum Zeitpunkt des PSA-Nadirs operiert worden waren [13], stießen wir auf einen anderen Wirkmechanismus als eine reine Zytoreduktion, über den die lokale Tumortherapie auch bei späterer Metastasierung das Überleben möglicherweise verlängern kann, nämlich die Verzögerung oder die Verhinderung der Resistenz gegenüber antiandrogenen Therapiemaßnahmen [22].

In einer ersten Analyse konnten 115 Patienten mit lokal fortgeschrittenem T4-Prostatakarzinom identifiziert werden, die in unserem Zentrum nach einer antiandrogenen Vorbehandlung zwischen 2000 und 2014 radikal prostatektomiert worden waren [13, 22]. Die Patientencharakteristika sind in Tab. 1 im Detail aufgeführt. Der PSA-Wert bei Diagnosestellung lag im Mittel bei 37,6 (Range: 2,44–282) ng/ml, zum Zeitpunkt der Operation im Mittel bei 0,69 (Range: 0,01–34,3) ng/ml. Die mediane Dauer der antiandrogenen Therapie, welche mit LHRH-Agonisten mit oder ohne zusätzlichen Androgenrezeptorantagonisten durchgeführt wurde, betrug 6 (Range: 2–20) Monate. Behandelt wurde in der Regel bis zum PSA-Nadir, wobei kein bestimmter absoluter oder relativer Grenzwert hinsichtlich des PSA-Abfalls vor Operation vorausgesetzt wurde. Die Reevaluierung der Operabilität nach induktiver Androgendeprivationstherapie erfolgte rein mittels digital-rektaler Untersuchung und transrektaler Sonographie, eine erneute Schnittbildgebung wurde nicht durchgeführt. In den vor Einleitung der induktiven ADT durchgeführten Staging-Untersuchungen (Skelettszintigraphie und CT Abdomen/Becken) waren keine Fernmetastasen nachgewiesen worden.

**Table 1:** 

Variable	Gesamt *n* = 115
*Alter (Jahre; Median [Range])*	66 (50–76)
*Initiales PSA (ng/ml; Median [Range])*	37,6 (2,44–282)
*Gleason-Score (Biopsie)*	
≤ 7	42 (36 %)
> 7	60 (52 %)
Unbekannt	14 (12 %)
*Dauer der induktiven ADT (Monate; Median [Range])*	6 (2–20)
*Präoperatives PSA (ng/ml; Median [Range])*	0,69 (0,01–34,3)
*TNM-Klassifikation, pT-Stadium im RP-Präparat*	
pT0–pT2	22 (19,1 %)
pT3	89 (77,4 %)
pT4	4 (3,5 %)
*Positive Schnittränder (R1)*	46 (40 %)
*Lymphknotenmetastasen (pN1)*	38 (33 %)
*R1 und pN1*	26 (22,6 %)

*ADT* Androgendeprivationstherapie, *PSA* prostataspezifisches Antigen, *RP* radikale Prostatektomie

Die erste Hälfte der Patienten war bis 2006 offen operiert worden, die zweite Hälfte ab 2006 ausschließlich laparoskopisch-roboterassistiert, wobei beide Gruppen sich bezüglich des sehr niedrigen Komplikationsrisikos nicht wesentlich voneinander unterschieden. Die gravierendsten intraoperativen Komplikation waren drei Rektumverletzungen (zwei in der offenen operierten Gruppe, eine in der roboterassistierten Gruppe), die allesamt intraoperativ erkannt und übernäht wurden, ohne dass sich daraus postoperative Komplikationen oder Interventionsnotwendigkeiten ergeben hätten. Komplikationen > Clavien-Dindo Grad 3 wurden nicht beobachtet. Bei allen Patienten wurde im Rahmen der radikalen Prostatektomie auch eine pelvine Lymphadenektomie durchgeführt, eine nerverhaltende Operation wurde den Patienten bei diesen Hochrisikobefunden nicht angeboten. Die Kontinenzergebnisse dieser Patienten wurden nicht systematisch ausgewertet. Daher kann man anhand der Patienten, die sich zur Nachsorge und Weiterbehandlung in unserer Klinik vorstellten, nur mit aller Vorsicht den Eindruck berichten, dass diese im Vergleich zu Patienten mit organbegrenztem Ausgangsbefund zumindest nicht schlechter zu sein scheinen.

Im Langzeitverlauf (hierfür lagen uns Follow-up-Daten von 111 Patienten vor) blieb der PSA-Wert bei nur 25 Patienten (22,5 %) im nicht nachweisbaren Bereich, von denen aber nur 19 nicht unter Hormontherapie standen. Den übrigen 6 Patienten hatte man aufgrund ungünstiger Ausgangsbefunde eine kontinuierliche Hormontherapie empfohlen. Im strengen Sinn der Definition lag das therapie- und rezidivfreie Überleben also bei etwa 17 %. Das schlechte rezidivfreie Überleben hatte aber keinerlei Einfluss auf das tumorspezifische Überleben oder das Gesamtüberleben: Das tumorspezifische Überleben aller 111 Patienten, für die ein Langzeitverlauf ermittelt werden konnte, lag bei 82 % nach 150 Monaten bei einem medianen Gesamtüberleben von 156 Monaten.

Diese Erstanalyse dieser Patienten zeigte also drei wesentliche Ergebnisse:

1. Prostatakarzinome im Stadium T4 sprechen sehr zuverlässig auf eine induktive Hormontherapie an und können fast alle zum Zeitpunkt des PSA-Nadirs nach 6–7 Monaten komplikationsarm und mit guten funktionellen Ergebnissen, zumindest die Kontinenz betreffend, prostatektomiert werden.
2. Weniger als 20 % dieser Patienten sind durch die Operation alleine definitiv geheilt, gemessen an der biochemischen Rezidivfreiheitsrate.
3. Die Langzeitüberlebensraten, auch über 15 Jahre hinaus, sind erstaunlich gut und liegen weit oberhalb der Rezidivfreiheitsrate von knapp 20 %. Diese breit klaffende Differenz zwischen den „echten“, biochemisch definierten Heilungsraten und den langfristigen Überlebensraten ist fraglos das bemerkenswerteste Ergebnis dieser Analyse.

**Figure 1:**
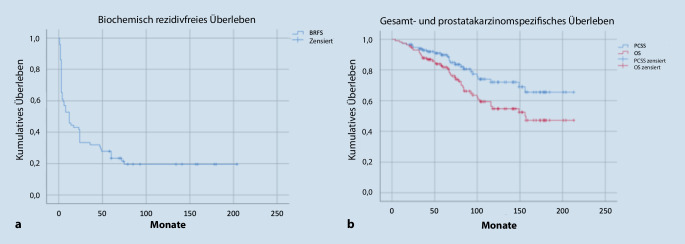

Eine zweite Analyse der Patientenverläufe fokussierte dementsprechend auf den Langzeitverlauf der Patienten, die bewiesenermaßen von ihrem ursprünglich weit fortgeschrittenen Tumor nicht geheilt waren, trotzdem aber langfristig nicht am Tumor verstorben sind. Einige dieser Patienten leben inzwischen fast 20 Jahre mit ihrem Tumor. Diese Daten konnten kürzlich publiziert werden [22]. Die Kernergebnisse der ersten Arbeit wurden im Wesentlichen bestätigt: Die biochemische Rezidivfreiheitsrate der Gesamtkohorte lag nach 200 Monaten bei etwa 20 %, die prostatakarzinomspezifische Überlebensrate bei 65 % und die Gesamtüberlebensrate bei 47 %, bei einem medianen Gesamtüberleben von 156 Monaten (Abb. 1).

Von der Gesamtgruppe aller 115 Patienten waren zum Zeitpunkt dieser Auswertung 44 verstorben. Die mediane Zeit bis zum Tod betrug 67 Monate. 19 dieser Todesfälle (43,1 %) waren tumorunabhängig nach einer medianen Zeit von 75 Monaten aufgetreten. Von 21 Patienten ohne PSA-Rezidiv ist bisher keiner tumorbedingt verstorben. Die übrigen 84 Patienten hatten während ihres weiteren Lebens ein PSA-Rezidiv. Der genaue Krankheitsverlauf dieser somit bewiesenermaßen nicht geheilten Patienten sollte genauer analysiert werden: Das tumorspezifische und das Gesamtüberleben dieser 84 Patienten lag nach 150 Monaten bei 61 % bzw. 44 %. Von den 84 Patienten waren nach durchschnittlich 95 Monaten Nachbeobachtungszeit noch 47 am Leben. 31 von ihnen, also ungefähr zwei Drittel, standen immer noch unter einer Standardhormontherapie. Nur 13 hatten eine Resistenz gegen die primäre Hormontherapie entwickelt und deswegen eine tertiäre Hormontherapie erhalten, auf die sie teilweise aber auch wieder langfristig sensibel blieben. Extrembeispiel ist ein Patient, der im kastrationsresistenten Stadium nach Erreichen eines PSA-Wertes von > 20 ng/ml eine Monotherapie mit Zytiga erhielt und darunter seit mehr als 6 Jahren ein nicht nachweisbares PSA aufweist. Seine Prostatektomie mit Nachweis von 4 befallenen Lymphknoten liegt inzwischen 17 Jahre zurück. Ein anderes Extrembeispiel für eine langanhaltende Empfindlichkeit auf die primäre Therapie ist ein Patient, der sich 2004 mit einem T4-Prostatakarzinom und einem PSA-Wert oberhalb von 250 ng/ml vorgestellt hatte. Er war im Juli 2005 bei einem PSA-Nadir von 0,45 ng/ml prostatektomiert worden. Histologisch zeigte sich ein Tumorstadium pT3a mit multiplen positiven Resektionsrändern ohne Lymphknotenbefall. Aufgrund der ungünstigen Ausgangssituation war ihm eine direkte Weiterführung der Hormontherapie empfohlen worden. Nachdem er darunter bis 2010 ein nicht nachweisbares PSA hatte, hat er sich selbst dazu entschlossen, sowohl die Hormontherapie wie auch jede Nachsorge zu beenden. Er stellte sich dann im September 2015 mit einem PSA-Wert oberhalb von 8500 ng/ml und diffusen Knochenmetastasen vor. Er wurde als vermeintlich moribunder Patient stationär aufgenommen und beschreibt selbst, dass er nach Verabreichung einer LHRH-Spritze innerhalb von Stunden eine drastische Verbesserung seines Allgemeinzustandes registriert hat. Der Patient konnte die Klinik 3 Tage später wieder verlassen und zeigte anschließend einen kontinuierlichen Abfall seines PSA-Wertes, der schlussendlich im August 2020 wieder den nicht nachweisbaren Bereich erreicht hat. Im Februar 2021 wurde der PSA-Wert mit 0,04 ng/ml erstmals wieder messbar, im Mai 2021 lag er bei 0,05 ng/ml.

## Diskussion

Das Konzept der radikalen Prostatektomie lokal weit fortgeschrittener Tumoren nach induktiver Hormontherapie geht auf die schon Jahrzehnte zurückreichende kasuistische Erfahrung zurück, dass diese weit fortgeschrittenen Prostatakarzinome nach Erreichen des PSA-Nadirs, der fast gesetzmäßig nach 6–7 Monaten eintritt, mit hoher Zuverlässigkeit und kaum erhöhtem Komplikationsrisiko operabel sind [30]. Die Rückgewinnung der Operabilität funktioniert sehr zuverlässig, unserer Homburger Arbeitsgruppe sind aus den letzten 20 Jahren keine Patienten erinnerlich, die man nach der Induktion nicht hätte operieren können, auch wenn solche Patienten nicht von Anfang an systematisch erfasst worden sind. Schon 1995 [30] war allerdings aufgefallen, dass nur die wenigsten Patienten durch diese Operationen wirklich geheilt werden konnten, zumindest, wenn man Heilung als nicht nachweisbares PSA ohne Hormontherapie definiert. Über die Jahrzehnte hinweg waren betroffene Patienten daher auch immer sehr zurückhaltend über den positiven Nutzen solcher Operationen aufgeklärt worden. Die Patienten wussten, dass das Komplikationsrisiko zwar nicht sonderlich groß war, dass andererseits aber die Chance, wirklich geheilt zu werden, die 20 %-Marke kaum überschreiten dürfte.

Nach 2015 fielen uns dann zunehmend mehr Patienten auf, die in dieser Konstellation überraschend lange am Leben geblieben waren, teilweise über 15 Jahre hinaus. Dies war Anlass, den Langzeitverlauf der Gesamtserie dieser Patientengruppe systematisch aufzuarbeiten und zu untersuchen, welchen Einfluss eine Lokaltherapie im Sinne einer radikalen Prostatektomie auf das Ansprechdauer einer späteren antihormonellen Therapie bei solchen Patienten haben könnte.

Bis vor etwa 20 Jahren war es unbestrittenes Lehrbuchwissen, dass ein metastasiertes Prostatakarzinom unter Hormontherapie nach ca. 18 Monaten hormonrefraktär zu werden beginnt und dass zu diesem Zeitpunkt die weitere Lebenserwartung der Patienten noch bei etwa 1 bis 1,5 Jahren liegt. Inzwischen mehren sich klinische Daten, die dafürsprechen, dass es jenseits der medikamentösen Behandlungsmöglichkeiten weitere Einflussfaktoren gibt, die die rezidivfreie und die tumorspezifische Überlebenszeit der Patienten verlängern können, insbesondere eine vor, vielleicht auch nach dem Eintritt der Metastasierung durchgeführte Prostatektomie. Die von uns gezeigten Daten suggerieren, dass die Primärtumorbehandlung zumindest bei den beschriebenen Begleitumständen in der Lage zu sein scheint, den Eintritt der Hormonresistenz hinauszuzögern und in Einzelfällen vielleicht auch ganz zu verhindern.

Die Hypothese einer Blockade der Hormonresistenz könnte auch die verfügbaren Daten zur „adjuvanten“ ADT des lymphknotenpositiven Prostatakarzinoms in ein anderes Licht tauchen. Messing et al. hatten bei solchen Patienten einen erstaunlichen tumorspezifischen Überlebensvorteil für adjuvant hormontherapierte im Vergleich zu nur prostatektomierten Patienten gezeigt [20, 21]: In der kombiniert behandelten Patientengruppe waren 17/47 Patienten verstorben, davon aber nur 7 am Prostatakarzinom. In der nur prostatektomierten Gruppe waren 28/51 verstorben, davon 20 am Prostatakarzinom. Die adjuvante Hormontherapie hat in dieser als prognostisch ungünstig eingestuften mikrometastatischen Situation das tumorbedingte Sterberisiko also um fast zwei Drittel reduziert. Es gibt nur sehr wenige Beispiele für einen so starken Therapieeffekt durch adjuvante Therapiemaßnahmen.

Dass die ADT allein nicht in der Lage ist, einen so drastischen Therapieeffekt bei pN+-Patienten auszulösen, belegen Daten von Bhindi et al., die für solche Patienten in einer sehr sorgfältig gematchten retrospektiven Studie die Kombination aus Prostatektomie und ADT mit der ADT allein (Verzicht auf Prostatektomie nach Nachweis von Lymphknotenmetastasen im Schnellschnitt) verglichen haben: Hierbei war das tumorspezifische Sterberisiko durch die Kombinationsbehandlung in ähnlich deutlicher Weise reduziert, nämlich um 50 % [6]. Offensichtlich kann die Kombinationsbehandlung die Effekte der beiden Einzeltherapien potenzieren, was neuerlich die Hypothese eines modulierenden Effektes der Primärtumorbehandlung auf die Entwicklung der Hormonresistenz zu untermauern scheint.

Damit stellt sich die Frage, ob auch bei bereits vorhandener Metastasierung die nachträgliche Entfernung des Primärtumors mit einer Überlebensverlängerung bei den betroffenen Patienten einhergehen könnte. Es wurden hierzu eine Reihe von retrospektiven Fallserien veröffentlicht, die allesamt zumindest einen positiven Trend zugunsten einer Primärtumortherapie beschrieben [3, 7, 11, 14]. Auch in einer sekundären Analyse der CHAARTED-Studie konnte dies bestätigt werden [1]. Eine klare Hypothese, warum eine radikale Prostatektomie für den Patienten in einer solchen klinischen Situation prognoseverbessernd sein könnte, hatte man nicht. In Analogie zum Nierentumor sprach man daher in diesem Kontext gerne von „zytoreduktiven“ Prostatektomien. Solche mechanistischen Konzepte („wenn man die Tumormasse um 30 % reduziert, steigt dafür die Lebenserwartung um 30 %“) haben sich in der langen Historie der Tumortherapie seltenst bewahrheitet. Die kasuistischen Erfahrungen, die viele Kliniken inzwischen mit solchen Operationen gesammelt haben, sind auch eher ernüchternd: Auch wenn die Operationen nicht sonderlich komplikationsträchtig sind, überzeugen die onkologischen Ergebnisse offensichtlich so wenig, dass selbst eine prospektive systematische Studie nicht gerechtfertigt erscheint. Im Gegensatz hierzu liegen uns seit 2018 mit der STAMPEDE-Studie randomisierte prospektive Daten zu einem positiven Effekt der Radiotherapie des Primärtumors auf das Gesamtüberleben bei Patienten mit geringer Metastasenlast (weniger als vier Knochenmetastasen und fehlende viszerale Metastasen) vor [23]. Eine kürzlich publizierte retrospektive Analyse aus dem LoMP-Register („local treatment of metastatic prostate cancer“) konnte zeigen, dass sich mit einer radikalen Prostatektomie bei Patienten mit „low volume“ metastasiertem Prostatakarzinom ein ähnliches Gesamtüberleben und prostatakarzinomspezifisches Überleben wie mit einer Radiotherapie des Primärtumors erreichen lässt [19].

**Figure 2:**
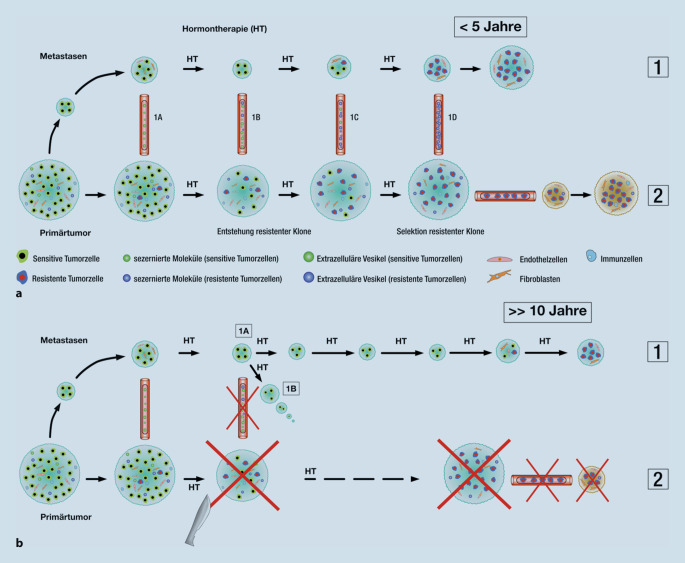

Über die molekularen Mechanismen, die einer solchen positiven Beeinflussung des weiteren Krankheitsverlaufs und insbesondere des Ansprechens auf eine Hormontherapie durch Primärtumorentfernung zugrunde liegen, konnte man bisher also nur spekulieren (Abb. 2).

Eine mögliche Erklärung wäre darin zu sehen, dass sich im Primärtumor durch die Interaktion der Prostatakarzinomzellen mit den verschiedenen zellulären und extrazellulären Komponenten des sehr speziellen intraprostatischen Mikromilieus einzelne Tumorklone ausbilden, die die Fähigkeit zur Entwicklung einer Kastrationsresistenz bereits in sich tragen. Die Interaktion mit der lokalen Mikroumgebung, insbesondere mit tumorassoziierten Fibroblasten, beeinflusst die Biologie der Prostatakarzinomzellen, wie wir in eigenen Arbeiten zeigen konnten [16]. Darüber hinaus können diese Fibroblasten auch die Therapieresistenz der Tumorzellen regulieren, wie in anderen Tumorentitäten belegt werden konnte [9]. Grundsätzlich weisen Primärtumoren eine komplexere klonale Heterogenität auf als Metastasen, zumindest in frühen Stadien. Und gerade das Prostatakarzinom wurde als sehr heterogener, multifokaler Tumor charakterisiert [12, 18]. Es ist anzunehmen, dass Zellklone, die eine Resistenz entwickeln, unter einer Hormontherapie expandieren oder zumindest erhalten bleiben. Aus unseren Beobachtungen lässt sich schließen, dass der Primärtumor Einfluss auf das Ansprechen der Metastasen nimmt, also die Resistenz induziert oder verstärkt.

Dies kann durch die systemische interzelluläre Kommunikation zwischen Primärtumor und Metastase erfolgen, vermittelt durch lösliche Mediatoren oder auch über extrazelluläre Vesikel, deren funktionelle Rolle bei urologischen Tumoren und für die Entstehung von Therapieresistenzen gezeigt werden konnte [16]. Darüber hinaus muss man auch davon ausgehen, dass verbliebene, therapieresistente Klone neue, eben auch therapieresistente Metastasen bilden. Basierend auf diesen Hypothesen würde man durch die Primärtumorentfernung die Hormonsensitivität der Metastasen, zumindest für einen längeren Zeitraum, erhalten.

Vermutlich sind die hier formulierten Hypothesen nur ein Teil der Wahrheit, und die molekularen Mechanismen, die dem Effekt einer Primärtumorentfernung auf die Hormonsensitivität des metastasierten Prostatakarzinoms zugrunde liegen, sind noch deutlich komplexer, als wir es uns in unseren Gedankenmodellen vorzustellen versuchen. Darauf weisen einige Publikationen bereits hin, die verschiedene, komplexe Modelle der Metastasierung und Therapieresistenz i. Allg. und speziell beim Prostatakarzinom beschreiben [12, 29]. Insbesondere muss man sicher auch weiter zwischen Lymphknotenmetastasen, die offensichtlich ausgesprochen hormonsensitiv sind, und Fernmetastasen unterscheiden [21], denn der Anteil lymphknotenpositiver Patienten in der von uns beschriebenen Serie war deutlich niedriger als man es vom klinischen Ausgangsstadium her erwarten würde. Eine große Herausforderung bei der Aufarbeitung der beteiligten molekularen Mechanismen besteht darin, die Primärtumorentfernung adäquat in einem präklinischen Modell abzubilden, um einzelne biologische Prozesse und potenziell involvierte Faktoren gezielt untersuchen zu können. Hier ist es uns kürzlich unter Verwendung eines in unserer Klinik etablierten orthotopen Xenograftmodells [17] gelungen, chirurgische Primärtumorentfernungen bei Mäusen mit oligometastasiertem Prostatakarzinom erfolgreich durchzuführen und zu zeigen, dass diese Primärtumorentfernung gegenüber einer Scheinoperation zu einer signifikant langsameren PSA-Progression sowie einem signifikant längeren Überleben der entsprechenden Tiere führt (Linxweiler et al., „under review“). In einem nächsten Schritt werden die Tiere zusätzlich zur Primärtumorentfernung eine Hormontherapie erhalten, um die oben erläuterten Hypothesen zu überprüfen. Die weitere Aufklärung der involvierten biologischen Prozesse könnte in Zukunft ein noch gezielteres therapeutisches Eingreifen ermöglichen, um uns dem Ziel der Verhinderung einer Kastrationsresistenz ein Stück näher zu bringen.

## Fazit für die Praxis

- Der natürliche Krankheitsverlauf des metastasierten Prostatakarzinoms unter Hormontherapie schien mit Entwicklung einer Hormonresistenz nach 12–18 Monaten und tumorbedingtem Tod der Patienten nach 2–3 Jahren für Jahrzehnte kaum beeinflussbar.
- In den letzten 20 Jahren hat sich die Prognose dieser Patienten wesentlich verbessert, zum einen durch die Einführung neuer, das Überleben verlängernder Medikamente, zum anderen dadurch, dass ein größerer Anteil an Patienten vor Eintritt der Metastasierung prostatektomiert war, und dass solche vorgängig prostatektomierten Patienten nach Eintritt der Metastasierung länger am Leben zu bleiben scheinen.
- Die Langzeitnachbeobachtung an unserer Klinik nach induktiver Hormontherapie bei T4-Karzinomen prostatektomierter Patienten, welche später ein Rezidiv entwickelt haben, zeigt ein unerwartet langes Ansprechen auf eine Androgendeprivationstherapie, was den vorteilhaften Effekt einer Prostatektomie auf die Prognose von Fernmetastasen erklären könnte.
- Damit postulieren wir, dass die Entfernung des Primärtumors zur Verhinderung einer Kastrationsresistenz oder zumindest zu einem längeren und besseren Ansprechen auf eine Hormontherapie beitragen kann.
- Die molekularen Mechanismen, die diesem Effekt einer radikalen Prostatektomie zugrunde liegen, sind bisher weitgehend unbekannt und sollen in zukünftigen präklinischen und klinischen Studien eingehender untersucht werden.
